# Identification of Zika virus in immature phases of *Aedes aegypti* and *Aedes albopictus*: a surveillance strategy for outbreak anticipation

**DOI:** 10.1590/1414-431X20198339

**Published:** 2019-11-07

**Authors:** V.C. Maniero, P.S.C. Rangel, L.M.C. Coelho, C.S.B. Silva, R.S. Aguiar, C.C. Lamas, S.V. Cardozo

**Affiliations:** 1 Departamento de Saúde, Programa de Pós-graduação em Biomedicina Translacional, Universidade do Grande Rio, Duque de Caxias, RJ, Brasil; 2 Departamento de Saúde, Faculdade de Medicina Veterinária, Universidade do Grande Rio, Duque de Caxias, RJ, Brasil; 3 Departamento de Saúde, Faculdade de Medicina, Universidade do Grande Rio, Duque de Caxias, RJ, Brasil; 4 Departamento de Genética, Laboratório de Virologia Molecular, Universidade Federal do Rio de Janeiro, Rio de Janeiro, RJ, Brasil; 5 Instituto Nacional de Infectologia Evandro Chagas, Fundação Oswaldo Cruz, Rio de Janeiro, RJ, Brasil; 6 Instituto Nacional de Cardiologia, Rio de Janeiro, RJ, Brasil

**Keywords:** Zika virus, Aedes aegypti, Aedes albopictus, Arbovirus, Georeferencing, Xenomonitoring

## Abstract

A progressive increase in the circulation of arboviruses in tropical countries has been observed, accounting for 700,000 yearly deaths in the world. The main objective of this article was to identify the presence of Zika (ZIKV), dengue (DENV), and Chikungunya (CHIKV) viruses in immature stages of *Aedes aegypti* and *Ae. albopictus*. Household collections of immature phases of the vectors were carried out in the years 2015 and 2016. A total of 2902 dwellings were visited and the rate of infestation with larvae and pupae of *Aedes* mosquitoes was 283/1462 (19.4%) in March 2015 and 55/1440 (3.8%) in June 2015. In March 2015, 907 larvae/pupae were collected (583 or 64.3% of *Ae. aegypti* and 324 or 35.7% of *Ae. albopictus*) while in June 2015 there was a reduction in the number of immature forms found: 197 larvae/pupae (121 or 61.4% of *Ae. aegypti* and 76 or 38.6% of *Ae. albopictus*). This reduction was accompanied by a decrease in suspected human ZIKV cases from March to June 2015. The RT-qPCR performed in 18 pools identified that three (two of *Ae. aegypti* and one of *Ae. albopictus*) were positive for ZIKV, and none were positive for DENV or CHIKV. Our findings demonstrated that ZIKV was present in immature stages of insect vectors in the study region at least five months prior to the peak of ZIKV associated cases. Xenomonitoring of immature phases of the vectors may prove useful for predicting outbreaks.

## Introduction

Vector-borne diseases account for about 17% of the total burden of communicable diseases and 700,000 deaths each year (1). In Brazil, dengue viruses (DENV) resurged in 1981 and since then, the country has been considered endemic for this arbovirus (2). In 2014, Chikungunya (CHIKV) and Zika (ZIKV) viruses, two exotic arboviruses, were introduced in Brazil causing epidemics with rapid dispersion in different states (3). The increasing outbreaks of ZIKV due to vector dispersion and lack of effective control and prevention actions led to a global public health concern (4). The most alarming clinical manifestations of ZIKV were cases associated with Guillain-Barré syndrome in adults (5) and the congenital ZIKV syndrome (6). Furthermore, reports of outbreaks of CHIKV emphasize the magnitude of its epidemics that accounted for 11,102 probable cases in 2017 and 4,861 cases by the middle of 2018 in the Southeast of Brazil (7). Adverse outcomes of CHIKV include neurological disease in the acute phase, and longstanding arthritis and myalgia. The acute phase of the three arboviral diseases may be clinically undistinguishable. Therefore, diseases caused by these viruses are of great public health relevance, but the epidemiological features in areas where the three viruses co-circulate are still not fully understood.

*Aedes aegypti* has competence for the transmission and is the main vector of all three arboviruses (8). In 2002, in an attempt to eradicate dengue, the Brazilian government created the National Dengue Control Program (PNCD). This program promoted initiatives aimed at surveillance and vector control measures, as well as environmental sanitation actions. The strategies consisted of identifying key areas using the Larval Index Rapid Assessment (LIRA) for *Ae. aegypti* and *Ae. albopictus*, and the spray application of the larvicide *Bacillus thuringiensis israelensis* (Bti) (9) in households that contained foci of immature mosquitoes. The LIRA is performed by sampling the vector within the municipalities, since the procedure quickly identifies the vector infestation rates. This method allows for the quick assessment of percentage figures and identifies predominant breeding sites, allowing rapid action for vector control (10).

Xenomonitoring carried out in immature stages of *Aedes* mosquitoes is considered a suitable tool in the early detection of viral circulation, predicting the possibility of outbreaks and epidemics (11). Performance of RT-PCR is the most used assay to identify the presence of virus in these vectors (12). In addition, other molecular techniques are applied as the sequencing of regions of the viruses to identify the circulating strain through phylogenetic analyses (13).

The objective of this study was to identify the presence of ZIKV, DENV, and CHIKV in the immature stages of *Ae. aegypti* and *Ae. albopictus* and georeferencing the outbreaks of these vectors in the fourth district (Xerem) of Duque de Caxias, Brazil.

## Material and Methods

### Case definition and study area

The city of Duque de Caxias has a Human Development Index of 0.711 and is divided into four districts: Duque de Caxias, Campos Eliseos, Imbariê, and Xerem. Xerem is located within the geographic coordinates 22°34'55.7"S 43°18'16.1"W and was our study area. It is a semi-rural district with 21,880 inhabitants, and it has 8000 households distributed in the urbanized area and its residents are provided prehospital care at the Álvaro Santos S. Figueira Unit, which is the reference unit in basic health care where about 300 patients per day are seen. In this unit, 28,064 records of patients were identified, photographed, and filed in a computer system.

Cases were randomly selected to sample 15 days of each month, distributed along all 4 weeks. Patients who did not live locally were excluded. In the analysis, all those who presented without fever or with a febrile illness with no identifiable source (respiratory, urinary, abdominal, soft tissue), a pruritic rash and/or non-purulent conjunctivitis were included in the study as suspected ZIKV cases. All illegible or incomplete medical records were excluded from the study.

Data on home address and clinical features were collected. The signs and symptoms obtained from the medical records were those suggested by the Ministry of Health to classify suspected ZIKV cases (14). Any other related description in the medical record, such as systemic events, were added in a separate field, in free text form. Skin color was not identified and diagnostic laboratory test results were not available. Patient information was encrypted to ensure patient confidentiality.

### House index (HI)

Collection of the immature stages of vectors from the domiciliary deposits was done, with subsequent georeferencing performed using the MECE^®^ system (Add Technologies, Brazil). MECE stands for strategic monitoring for epidemic control, which consists of a mobile device (smartphone) with information from the study area: planned route, route performed, classification of the home (closed, refused, vacant land, or inspected by work team), types of reservoirs/breeding sites of the vectors, and sample collection of the vectors.

We carried out the collection of immature mosquito stages for determination of HI in March and June of 2015 and the city health council provided data for 2016. These data were acquired with the MECE^®^ system and served as the basis for the georeferencing.

Systematic collection of immature forms in one in every five dwellings (following the guidelines of the National Guidelines for the Prevention and Control of Dengue Epidemics (10)) was carried out during the months of March and June 2015. The immature stages were stored in containers containing 70% alcohol and sent for identification in the laboratory, according to the dichotomous key of vectors (15).

HI was calculated from the data collected during the home visits, using the ratio of the number of positive properties and the number of properties surveyed expressed as a percentage. Thus, classification was based on the HI following the possibility of vector transmission, namely: satisfactory (HI<1%); alert status (HI between 1 and 3.9%); and at-risk (HI>3.9%) (10).

Precipitation and temperature indexes were obtained from March to June 2015 and 2016, through the portal <http://www.inmet.gov.br/portal/> of the National Institute of Meteorology (16). The mean temperature was calculated by the daily reference obtained from 0/12 h. The average rainfall was calculated by dividing the monthly precipitation by the number of rainy days.

The Research Ethics Committee of Universidade do Grande Rio approved the study (CAAE 54544316.3.0000.5283).

### Molecular analysis

Immature forms of the vectors were collected in bimonthly domiciliary visits from June 2015 to April 2016 (Supplementary Figure S1). The number of houses visited for collection of immature forms was 18 in 2015 and 9 in 2016. They were not the same dwellings over time. These dwellings were indicated to be the location of mosquito foci by local health agents.

The immature phases were kept viable in a container containing 10 mL of deionized water and sent for laboratory identification, following the dichotomous key of vectors (15). After identification, these immature phases were washed twice by immersion in deionized water and separated in pools, according to the species, evolutionary phase (larva or pupa), and month of collection. Each pool had 1 to 137 specimens. They were then frozen at –80°C, still alive and without any viral additive (17), where they remained until the RNA extraction procedure was performed.

Viral RNA extraction was performed using the QIAamp MinElute Virus Spin Kit, (QIAGEN, Inc., USA), with appropriate adaptations for the immature phases of the vectors. In this case, they were immersed in 200 μL of phosphate-buffered saline (PBS) and 25 µL of protease and macerated with the Tissue Ruptor (QIAGEN, Inc.) until the whole solution became homogeneous. Afterwards, in order to stop protease digestion, the samples were incubated at 56°C for 15 min, and then centrifuged at 3,400 *g* for 10 min at room temperature. Subsequently, the supernatant was transferred to a new tube and the steps determined by the manufacturer were followed. The RNA quantification was performed by the spectrophotometer NanoPhotometer^®^ (Implen GmbH, Germany).The eluted RNAs were kept in a freezer at –80°C until further use (18) The reverse transcriptase reaction followed by real-time PCR (RT-qPCR) was performed to detect the presence of nucleic acid of ZIKV, DENV, and CHIKV in each pool containing viral RNA extracted. For this, we used a superscript III Platinum One-Step qRT-PCR System (Invitrogen, USA) containing reagents capable of performing the reverse transcription reaction using oligonucleotides specific for the target region generating a specific cDNA for the amplification region in the Applied Biosystems^®^ 7500 Real-Time Thermal Cycler (USA). In this methodology, specific probe and oligonucleotides are mixed in a solution containing all reagents necessary for amplification of the region of interest. The One-step RT-qPCR kit used was the TaqMan^®^ Fast Virus 1-Step Master Mix kit (Thermofisher Scientific, USA) with primers and probes specifically designed for DENV, ZIKV, and CHIKV (19-20) (Supplementary Table S1).

Cycle threshold values below 38.5 are considered positive according to CDC guidelines (20). All the RT-PCR reactions were performed in triplicates, including negative and positive controls. However, no sequencing data was possible due to lack of enough RNA in the samples to perform further DNA sequencing reactions.

### Treatment of outbreaks

Following the determination of the HI, an action plan to combat the adult vector of *Ae. aegypti* and *Ae. albopictus* was made. The plan involved scheduled treatments using an ultra-low volume electric insecticide nebulizer UVB^®^ (Add Technologies, Brazil). Additionally, infested foci were treated with the larvicide Bti (Valent Bio Sciences Corp., USA), in order to eliminate the immature stages of the vector directly in the foci, as recommended by the Brazilian Ministry of Health.

### Georeferencing

The maps were generated with the georeferenced data stored in the MECE*^®^* system, through the cartographic databases provided by the Brazilian Institute of Geography and Statistics (IBGE), with the use of the QGIS software (version 3.2.0; <qgis.org>). The number of ZIKV cases per 100,000 inhabitants was calculated from the total number of ZIKV cases in the census tract, divided by the total population resident in the census tract, multiplied by 100,000. The information regarding the population in the area of Xerem was obtained from the last census (21).

### Statistical analysis

The relationships between HI (*Ae. aegypti* and *Ae. albopictus*), rainfall, and temperature were determined by Pearson’s correlation analysis, with the aid of Bioestat^®^ package version 5.0 (Brazil). Descriptive analysis of clinical data was performed using the Microsoft Office Excel^®^ 2007 program (USA). The minimum infection rate (MIR) was calculated by dividing the number of pools infected by specimens by the total number of immature phases tested for each specimen, multiplied by 1,000 (22).

## Results

### Case definition

According to the analysis of the medical records, the total number of suspected cases for arboviruses in 2015 was 969 (4,296/100,000 inhabitants), and of these 146 (15.1%) were classified as caused by ZIKV, and in 2016, there were 2012 suspected cases (8,866/100,000 inhabitants) of which 777 (38.6%) were classified as ZIKV.

Although the symptoms of ZIKV among other arboviruses are very similar, the analyses showed that in the years 2015 and 2016, among 146 suspected ZIKV cases, two symptoms were the most frequent: rash occurred in 99 (67.8%) in 2015 and 614 (79.0 %) in 2016 and pruritus occurred in 93 (63.7%) in 2015 and 549 (70.7%) in 2016.

### Home visits and georeferencing

A total of 2902 dwellings were visited in the urban area of Xerem, and the rate of infestation with larval forms (larvae and pupae) of *Aedes* mosquitoes was 283/1462 (19.4%) in March 2015 and 55/1440 (3.8%) in June 2015. In March 2015, 907 larvae/pupae were collected (583 or 64.3% of *Ae. aegypti* and 324 or 35.7% *Ae. albopictus*) while in June 2015 there was a reduction in the number of immature forms found: 197 larvae/pupae (121 or 61.4% of *Ae. aegypti* and 76 or 38.6% of *Ae. albopictus)*.

**Figure 1:**
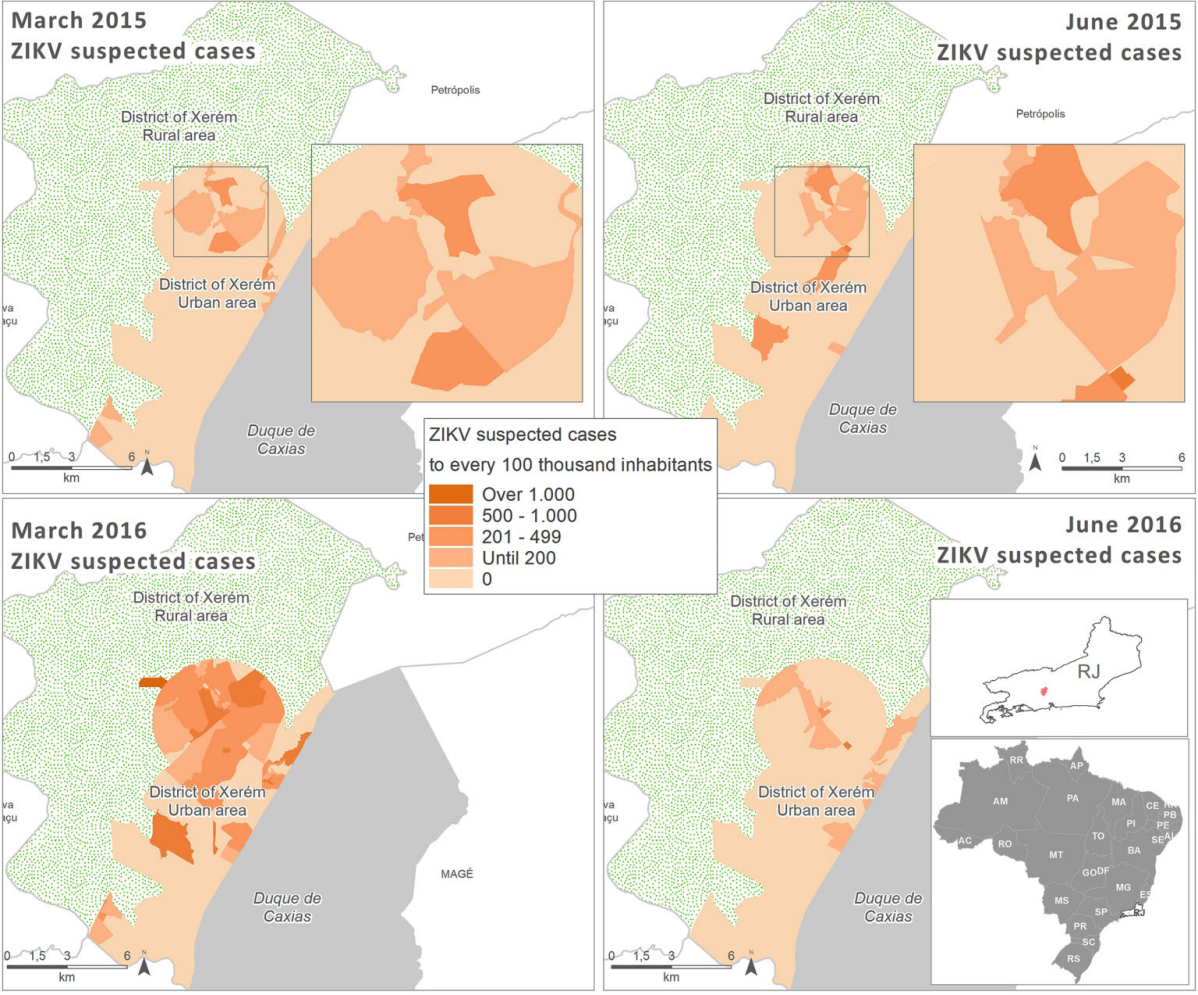
Spatial distribution of Zika virus (ZIKV) suspected cases in March and June 2015 and 2016 in Xerem, Duque de Caxias, Brazil.

Our data demonstrated that suspected ZIKV cases were concentrated in the urbanized area of Xerem. This area is more densely populated and has a high concentration of vectors (*Ae. aegypti* and *Ae. albopictus*). It is noteworthy that in 2015 there were already suspected cases of ZIKV in this region, and the number of cases reached its apex in March 2016, the period when the outbreak was characterized (Figure 1.).

**Figure 2.**
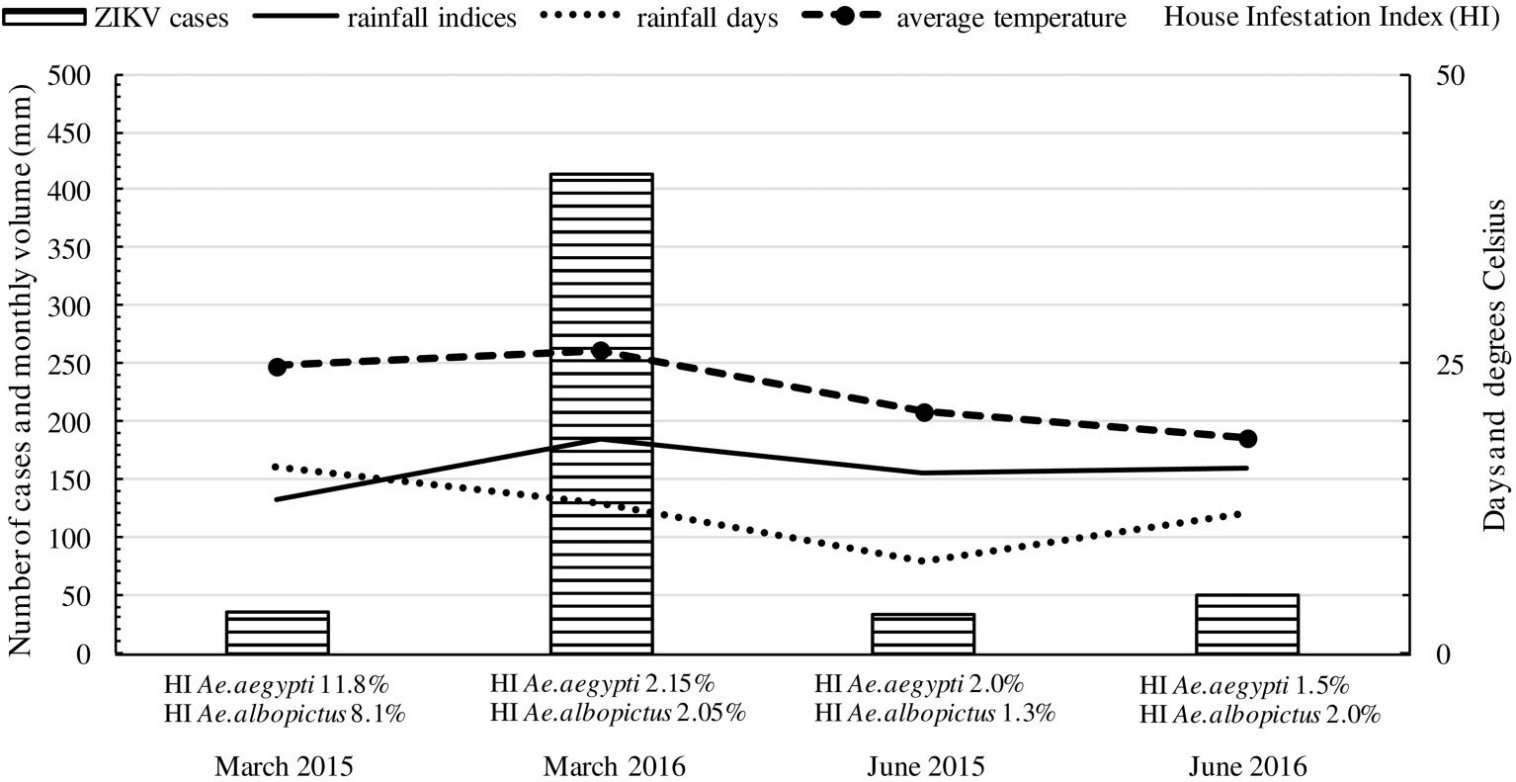
Relationship of number of Zika virus (ZIKV) suspected cases with rainfall and temperature data in the study months. HI: house index.

The HI in 2015 was high for both insect vectors, 11.8% for *Ae. aegypti* and 8.1% for *Ae. albopictus*. March was considered a month of heavy rainfall, as is expected during summer, with a total of 16 rainy days and the accumulated rainfall of 227 mm. In June, there were only three peaks of rainfall above 28 mm, with a total of 169 mm (Figure 2).

Average temperature in March was 24.7°C (maximum 31°C; minimum 20°C) and in June, 20.8°C (maximum 31°C; minimum 13°C). In March 2016, HI was 2.15% for *Ae. aegypti* and 2.05% for *Ae. albopictus*; rainfall index was high (Figure 2), and there were 777 ZIKV suspected cases, a considerable number.

A strong positive correlation was found between rainfall and HI, both for *Ae. aegypti* (r=0.931) and for *Ae. albopictus* (r=0.942); on the other hand, there was a moderate positive correlation between temperature and HI (r=0.457 and r=0.439 for each vector, respectively). These data show that, despite the important role of temperature in the development of the immature stages of the vectors, rainfall plays a more decisive role in this respect.

During the home visits, actions were taken to combat the vector, where the health agents applied Bti, in the deposits of immature stages. In the sites that presented HI> 3.99%, control measures were taken against the adult vector by use of an insecticide nebulization (UBV^®^ Electric, Fumajet, Add Technologies, Brazil).

### Detection of arbovirus in mosquito pools

A total of 615 immature stages (larvae and pupae) were collected every two months from June 2015 to April 2016, 442 (72%) of which were identified as *Ae. albopictus* and 173 (28%) as *Ae. aegypti*. The RT-qPCR for DENV, ZIKV, and CHIKV was performed in all pools and, of the 18 pools analyzed, 3 (which only had larvae) were positive for ZIKV, suggesting the occurrence of transovarian transmission of ZIKV of 11.6% (2/173) for *Ae. aegypti* and 2.3% (1/442) for *Ae. albopictus*. The positive pools were from October 2015, containing 38 *Ae. aegypti* and December 2015, containing 33 larvae of *Ae. albopictus* and 3 larvae of *Ae. aegypti* (Table 1). According to these results, the cycle thresholds of positive pools, when screened by RT-qPCR, were: in October/2015: 37.34 (larvae *Ae. aegypti*) and December/2015: 36.00 (larvae *Ae. albopictus*); 37.84 (larvae *Ae. aegypti*). CHIKV and DENV were not identified in any of the pools evaluated.

**Table 1.** Identification of dengue virus (DENV), Zika virus (ZIKV), and Chikungunya virus (CHIKV) in the immature stages of *Aedes aegypti* and *Ae. albopictus* by RT-qPCR.

Month/year	*Ae. aegypti*		*Ae. albopictus*		RT-qPCR		
	Pools		Pools		DENV	ZIKV	CHIKV
	Larvae	Pupae	Larvae	Pupae			
Jun/15	0	0	80	0	neg	neg	neg
Aug/15	75	0	0	0	neg	neg	neg
Oct/15	0	1	0	0	neg	neg	neg
Oct/15	38	0	0	0	neg	37.34	neg
Oct/15	0	0	137	0	neg	neg	neg
Oct/15	0	0	93	0	neg	neg	neg
Dec/15	3	0	0	0	neg	37.84	neg
Dec/15	0	0	33	0	neg	36.00	neg
Dec/15	0	6	0	0	neg	neg	neg
Feb/16	5	0	0	0	neg	neg	neg
Feb/16	0	6	0	0	neg	neg	neg
Feb/16	0	0	46	0	neg	neg	neg
Feb/16	0	0	0	4	neg	neg	neg
Apr/16	0	14	0	0	neg	neg	neg
Apr/16	25	0	0	0	neg	neg	neg
Apr/16	0	0	37	0	neg	neg	neg
Apr/16	0	0	0	6	neg	neg	neg
Apr/16	0	0	0	6	neg	neg	neg

## Discussion

Our results showed the identification of ZIKV in immature phases of the vectors *Ae. aegypti* and *Ae. albopictus*, preceding a large outbreak located in the study region, which occurred in March 2016. The use of molecular tools in entomological surveillance may contribute to an early detection of arboviruses, as indicated in some studies (12). This forecast may assist in the organization of human and material resources in epidemic periods.

Previous experimental studies have demonstrated that a number of mosquito-borne flavivirus pathogens are vertically transmitted in their insect vectors, providing a mechanism for these arboviruses to persist during adverse climatic conditions or in the absence of a susceptible vertebrate host. The transovarial transmission of arbovirus was recognized in the 1950s in studies carried out with DENV, being found in male specimens, that is, those that do not carry out hematophagy, in immature stages (larvae and pupae) of *Ae. aegypti*, with a MIR of 1:2067 for DENV-2 (23). Since then, several studies have been carried out in different regions, confirming these findings (24). More recently, a study demonstrated that *Ae. aegypti* and *Ae. albopictus* mosquitoes from Thailand were capable of transmitting CHIKV vertically in the laboratory; *Ae. albopictus* was more susceptible and had a greater ability to transmit the viruses vertically than *Ae. aegypti*. *Ae. aegypti* and *Ae. albopictus* mosquitoes were able to transmit CHIKV vertically to F5 and F6 progenies, respectively (24). However, in the present study, none of our pools yielded CHIKV or DENV, which may be related to the small number of immature stages tested associated to the fact that in 2015 and 2016 CHIKV and DENV were not epidemic in this area in Brazil (25).

Detection of arbovirus in immature stages or mosquitoes has been well described. In our study of 615 specimens distributed in 18 pools, we found 3 positive for ZIKV, with a MIR for *Ae. aegypti* of 11.6 (2/173) and for *Ae. albopictus* of 2.3 (1/442) suggesting that arboviruses have been transmitted by the transovarial route. Two other studies also obtained similar data, with immature phases developed until the adult forms, where DENV-4 was detected in *Ae. aegypti* and MIR was 10.5 (8/758) (26) and another in Fortaleza for DENV-3, which despite the high incidence rate of DENV, the MIR for *Ae. aegypti* was 0.5 (1/2005) and for *Ae. albopictus*, 9.4 (2/212) (27). In a study carried out in Colombia, using an immunofluorescent assay with a flavivirus-specific monoclonal antibody on adult *Ae. aegypti* females, the MIR was 11.6 (24/2065) for DENV but no vertical transmission of DENV could be detected in 1552 male *Ae. aegypti* collected (28).

Transovarial transmission of arbovirus allows persistence of the viruses in nature and is relevant to the epidemiological role in outbreaks of vector-borne diseases (29). Our data suggested that the transovarial transmission capacity of both *Ae. aegypti* and *Ae. albopictus*, may be related to the persistent survival of ZIKV during interepidemic periods, which has already been demonstrated by Li et al. (30). Interestingly, *Ae. albopictus* may play an important role in the maintenance of ZIKV throughout the year, as it was found persistently in foci in the studied area; it is a species more adapted to temperate climatic conditions and less urbanized areas (31).

A Brazilian study from an area not distant from ours showed the presence of ZIKV in nature, in the adult insect (mosquitoes). A total of 550 *Ae. aegypti* (315 females and 235 males, grouped in 198 pools), and 26 *Ae. albopictus* (20 and 6; 21 pools) were studied. Three pools of *Ae. aegypti* were positive for ZIKV, one of which had male mosquitoes; the natural infection of a male mosquito was interesting and strongly suggested vertical transmission, as only female mosquitoes are blood-sucking. These results reinforced ZIKV circulation in this region during this period, indicating the occurrence of the outbreak in the following year. The cycle thresholds of positive pools were similar to our study, and some pools contained very few specimens, which is also similar to our study. The difference between their study and ours is that we identified ZIKV in the immature forms of both *Aedes* species, showing that *Ae. albopictus* may also be a vector for ZIKV in Brazil (32). *Aedes albopictus* has been found naturally infected with the viruses in Gabon in 2007 (33) and in Mexico in 2016 (4), and has been shown to be experimentally infected in the laboratory in Florida, although with low efficiency (34). In another study, designed to test whether ZIKV could be vertically transmitted, female *Ae. aegypti* and *Ae. albopictus* were injected with ZIKV, and their F1 adult progeny were tested for ZIKV infection. Six of 69 *Ae. aegypti* pools, comprised of a total of 1738 F1 adults, yielded ZIKV upon culture, giving a minimum filial infection rate of 1:290. In contrast, none of 803 F1 *Ae. albopictus* adults (32 pools) yielded ZIKV (35). It is important to note that injecting mosquitoes does not replicate what occurs in nature, which is oral contamination via bites. In a study by Ciota et al. (36), adult insects fed orally with ZIKV in the laboratory had their progeny tested; of 104 *Ae. aegypti* pools, 6 were ZIKV positive, indicating a filial infection rate (FIR) of 11.9, a ratio of ≅1:84, which is substantially greater than that found in another study (35) as well as ratios historically measured for flaviviruses (4–8), and 1 of 17 pools of *Ae. albopictus* tested positive, which yielded a similar FIR (11.8 [range 1.7–134.8]).

In Brazil, arbovirus monitoring using RT-PCR in larvae of *Aedes* spp. is performed sporadically by some surveys (17) and is not used as a routine tool in arbovirus control programs. The RT-PCR has several advantages, such as rapid detection, sensitivity, and specificity, and can be applied in the surveillance of arboviruses (37), especially when negative results are obtained by other tests, such as viral isolation in cell cultures (38). Our results confirmed the high level of sensitivity by RT-PCR in pools containing a smaller amount of *Ae. aegypti*, as obtained in other studies (27,39).

Due to the ZIKV outbreak in Brazil, several cases of the congenital syndrome, mainly associated with microcephaly in newborns, have been reported with the detection of viral RNA in the amniotic fluid of the affected patients (6). The threat posed by ZIKV has great implications not only on immediate public health but also on family economy and social resources, due to the persistent long-term sequelae of the congenital ZIKV syndrome (40). Reliable and sensitive surveillance of these arboviruses, which includes a system for the detection of emerging pathogens, is of paramount importance in order to effectively manage future outbreaks.

Our findings demonstrated that ZIKV was present in immature stages of vector insects in the study region at least five months prior to the peak of ZIKV associated cases in March 2016. Data from molecular xenomonitoring of immature phases of the vectors may contribute to an early detection of arboviruses helping to plan human and material resources for potential outbreaks.

## Supplementary Material

Click here to view [pdf].
